# ITS2 data corroborate a monophyletic chlorophycean DO-group (Sphaeropleales)

**DOI:** 10.1186/1471-2148-8-218

**Published:** 2008-07-25

**Authors:** Alexander Keller, Tina Schleicher, Frank Förster, Benjamin Ruderisch, Thomas Dandekar, Tobias Müller, Matthias Wolf

**Affiliations:** 1 Department of Bioinformatics, University of Würzburg, Am Hubland, 97074 Würzburg, Germany

## Abstract

**Background:**

Within Chlorophyceae the ITS2 secondary structure shows an unbranched helix I, except for the '*Hydrodictyon*' and the '*Scenedesmus*' clade having a ramified first helix. The latter two are classified within the Sphaeropleales, characterised by directly opposed basal bodies in their flagellar apparatuses (DO-group). Previous studies could not resolve the taxonomic position of the '*Sphaeroplea*' clade within the Chlorophyceae without ambiguity and two pivotal questions remain open: (1) Is the DO-group monophyletic and (2) is a branched helix I an apomorphic feature of the DO-group? In the present study we analysed the secondary structure of three newly obtained ITS2 sequences classified within the '*Sphaeroplea*' clade and resolved sphaeroplealean relationships by applying different phylogenetic approaches based on a combined sequence-structure alignment.

**Results:**

The newly obtained ITS2 sequences of *Ankyra judayi, Atractomorpha porcata *and *Sphaeroplea annulina *of the '*Sphaeroplea*' clade do not show any branching in the secondary structure of their helix I. All applied phylogenetic methods highly support the '*Sphaeroplea*' clade as a sister group to the 'core Sphaeropleales'. Thus, the DO-group is monophyletic. Furthermore, based on characteristics in the sequence-structure alignment one is able to distinguish distinct lineages within the green algae.

**Conclusion:**

In green algae, a branched helix I in the secondary structure of the ITS2 evolves past the '*Sphaeroplea*' clade. A branched helix I is an apomorph characteristic within the monophyletic DO-group. Our results corroborate the fundamental relevance of including the secondary structure in sequence analysis and phylogenetics.

## Background

Taxonomists face inconsistent or even contradictory clues when they examine the affiliation of organisms to higher taxonomic groupings. Several characters may yield alternative hypotheses explaining their evolutionary background. This also applies to the taxonomic position of the Sphaeropleaceae [1-23]. Different authors affiliate the green algal family by morphological characters to either ulvophytes or chlorophytes, until amendatory Deason et al. [10] suggested that the Neochloridaceae, the Hydrodictyaceae and the Sphaeropleaceae should be grouped as Sphaeropleales within the chlorophytes, since all of them have motile biflagellate zoospores with a direct-opposite (DO) confirmation of basal bodies.

Subsequently, other taxonomic lineages (the '*Ankistrodesmus*' clade, the '*Bracteacoccus*' clade, the '*Pseudomuriella*' clade, *Pseudoschroederia*, the '*Scenedesmus*' clade, *Schroederia *and the '*Zofingiensis*' clade) were added to this biflagellate DO group, because they show molecular affiliation to either Neochloridaceae or Hydrodictyaceae [24].

Although nowadays most authors agree that the DO group is monophyletic, until now no study pinpointed the taxonomic linkage of the name-giving '*Sphaeroplea*' clade to the remaining 'core Sphaeropleales' persuasively with genetic evidence [6,23], i.e. the sister clade remains unclear [15,24]. Likewise, with respect to morphology, studies of 18S and 26S rRNA gene sequences neither resolve the basal branching patterns within the Chlorophyceae with high statistical power nor corroborate a monophyletic biflagellate DO group without ambiguity [6,23].

Müller et al. [25] obtained moderate statistical support for the close relationship of the '*Sphaeroplea*' clade and the 'core Sphaeropleales' with profile distances of 18S and 26S rDNA. In this study we followed and expanded their methodology with a very different phylogenetic marker. The internal transcribed spacer 2 (ITS2), the region of ribosomal RNA between the 5.8S rRNA gene and the large subunit (26S rDNA) has proven to be an appropriate marker for the study of small scale phylogenies of close relatives [26-29]. The sequence is in contrast to the bordering regions of ribosomal subunits evolutionary not conserved, thus genetic differentiation is detectable even in closely related groups of organisms. By contrast, the secondary structure seems to be well conserved and thus provides clues for higher taxonomic studies [27,30-33]. Secondary structure information is furthermore especially interesting within the Chlorophyceae, because van Hannen et al. [34] described an uncommon branching of ITS2 helix 1 within the genera *Desmodesmus*, *Hydrodictyon *[35] and *Scenedesmus*. It is not known when this feature evolved and whether it is, as we expect, an apomorphic feature for the DO-group. It is obvious that phylogenetic statements should be improvable by inclusion of structural information in common sequence analysis. For example, Grajales et al. [36] calculated morphometric matrices from ITS2 secondary structures for phylogenetic analyses, but treated information of sequence and structure as different markers. Here we combine sequence with structural information in just one analysis. Aside from the biological problem, we address the pivotal question of a methodological pipeline for sequence-structure phylogenetics using rDNA data.

## Methods

### DNA extraction, amplification and sequencing

Extraction of genomic DNA from cultured cells of *Ankyra judayi, Atractomorpha porcata *and *Sphaeroplea annulina *was done using Dynabeads^® ^(DNA DIRECT Universal, Dynal Biotech, Oslo, Norway) according to the manufacturer's protocol. PCR reactions were performed in a 50 μl reaction volume containing 25 μl FastStart PCR Master (Roche Applied Science), 5 μl gDNA and 300 nM of the primers ITS3 (5'-GCA TCG ATG AAG AAC GCA GC-3') and ITS4 (5'-TCC TCC GCT TAT TGA TAT GC-3') designed by White et al. [37].

Cycling conditions for amplification consisted of 94°C for 10 min, 30 cycles of 94°C for 30 s, 50°C for 30 s and 72°C for 45 s, followed by a final extension step of 10 min at 72°C. PCR products were analysed by 3% agarose gel electrophoresis and ethidium bromide staining.

PCR probes where purified with the PCR Purificaton Kit (Qiagen) and where quantified by spectrometry. Each sequencing probe was prepared in an 8 μl volume containing 20 ng DNA and 1.25 μM Primer. Sequencing was carried out using an annealing temperature of 50°C with the sequencer Applied Biosystems QST 3130 Genetic Analyzer by the Institute of Hygiene and Microbiology (Würzburg, Germany).

### ITS2 secondary structure prediction

ITS2 secondary structures of the three newly obtained sequences were folded with the help of RNAstructure [38] and afterwards manually corrected. All available 788 chlorophycean ITS2 sequences were obtained from the NCBI nucleotide database. The ITS2 secondary structure of *Atractomorpha porcata *was used as template for homology modelling. Homology modelling was performed by using the custom modelling option as provided with the ITS2-Database [30-33] (identity matrix and 50% threshold for the helix transfer). Forty-nine species representing the chlorophycean diversity were retained and used as comparative taxa in inferring phylogenies (Table 1). For this taxon sampling, accurate secondary structures of sequences were now folded by RNAstructure and additionally corrected using Pseudoviewer 3 [39]. We standardized start and end of all helices according to the optimal folding of the newly obtained sequences.

**Table 1:** Chlorophyte species used for this investigation.

Clade	Species	Strain	GenBank
'*Sphaeroplea *'	*Ankyra judayi *(G.M. Smith) Fott 1957	SAG 17.84	EU352800
	*Atractomorpha porcata *Hoffman 1984 strain	SAG 71.90	EU352803
	*Sphaeroplea annulina *(Roth) C. Agardh 1824	SAG 377.1a	EU352801
	*Sphaeroplea annulina *(Roth) C. Agardh 1824	SAG 377.1e	EU352802

'*Dunaliella*'	*Haematococcus droebakensis *Wollenweber 1908	-	U66981
	*Dunaliella parva *Lerche 1937	-	DQ116746
	*Dunaliella salina *(Dunal) Teodoresco 1905	CCAP 19/18	EF473746

'*Hydrodictyon *'	*Hydrodictyon africanum *Yamanouchi 1913	UTEX 782	AY779861
	*Hydrodictyon patenaeforme *Pocock	CCAP 236/3	AY577736
	*Hydrodictyon reticulatum *(Linnaeus) B. de St.-Vincent 1824	CBS	AY779862
	*Pediastrum braunii *Wartmann 1862	SAG 43.85	AY577756
	*Pediastrum duplex *Meyen 1829	UTEX 1364	AY779868
	*Pseudopediastrum boryanum *(Raciborski) Sulek 1969	UTEX 470	AY779866
	*Sorastrum spinulosum *Nägeli 1849	UTEX 2452	AY779872
	*Stauridium tetras *(Ehrenberg) Ralfs 1844	EL 0207 CT	AY577762

'*Oedogonium*'	*Bulbochaete hiloensis *(Nordstedt) Tiffany 1937	-	AY962677
	*Oedogonium cardiacum *(Hassall) Wittrock 1870	-	AY962675
	*Oedogonium nodulosum *Wittrock 1872	-	DQ078301
	*Oedogonium oblongum *Wittrock 1872	-	AY962681
	*Oedogonium undulatum *(Brébisson) A. Braun 1854	-	DQ178025

'*Reinhardtii*'	*Chlamydomonas incerta *Pascher 1927	SAG 81.72	AJ749625
	*Chlamydomonas komma *Skuja 1934	-	U66951
	*Chlamydomonas petasus *Ettl	SAG 11.45	AJ749615
	*Chlamydomonas reinhardtii *Dangeard 1888	CC-620	AJ749638
	*Chlamydomonas typica *Deason & Bold 1960	SAG 61.72	AJ749622
	*Eudorina elegans *Ehrenberg 1831	ASW 107	AF486524
	*Eudorina unicocca *G.M. Smith 1930	UTEX 1215	AF486525
	*Gonium octonarium *Pocock 1955	Tex	AF054424
	*Gonium pectorale *O.F. Müller 1773	Chile K	AF054440
	*Gonium quadratum *E. G. Pringsheim ex H. Nozaki	Cal 3-3	AF182430
	*Pandorina morum *(O.F. Müller) Bory de Saint-Vincent 1824	Chile	AF376737
	*Volvox dissipatrix *(Shaw) Printz	-	U67020
	*Volvox rousseletii *G.S.West	-	U67025
	*Volvulina steinii *Playfair 1915	-	U67034
	*Yamagishiella unicocca *(Rayburn & Starr) Nozaki 1992	ASW 05129	AF098181

'*Scenedesmus*'	*Desmodesmus abundans *(Kirchner) Hegewald 2000	UTEX 1358	AJ400494
	*Desmodesmus bicellularis *(Chodat) An, Friedl & Heg. 1999	CCAP 276/14	AJ400498
	*Desmodesmus communis *(Hegewald) Hegewald 2000	UTEX 76	AM410660
	*Desmodesmus elegans *(Hortobágyi) Heg. & Van. 2007	Heg 1976–28	AM228908
	*Desmodesmus opoliensis *(P.G. Richter) Hegewald 2000	EH 10	AM410655
	*Desmodesmus pleiomorphus *(Hindák) Hegewald 2000	UTEX 1591	AM410659
	*Desmodesmus quadricauda *(Turpin) Hegewald	-	AJ400495
	*Scenedesmus acuminatus *(Lagerheim) Chodat 1902	UTEX 415	AJ249511
	*Scenedesmus acutiformis *(B. Schröder) F. Hindák 1990	SAG 276.12	AJ237953
	*Scenedesmus basiliensis *Chodat 1926	UTEX 79	AJ400489
	*Scenedesmus dimorphus *(Turpin) Kützing 1833	UTEX 417	AJ400488
	*Scenedesmus longus *Meyen 1829 ex Ralfs	NIOO-MV5	AJ400506
	*Scenedesmus obliquus *(Turpin) Kützing 1833	Tow 9/21P-1W	DQ417568
	*Scenedesmus pectinatus *Meyen 1828	An 111a	AJ237954
	*Scenedesmus platydiscus *(G.M. Smith) Chodat 1926	UTEX 2457	AJ400491
	*Scenedesmus raciborskii *Woloszynska 1914	An 1996–5	AJ237952
	*Scenedesmus regularis *Svirenko	Heg 1998–2	AY170857
	*Scenedesmus wisconsinensis *(G.M. Smith) Chodat 1996	An 41	AJ237950

Listed is the current clade classification of the species [69,70,24] and the GenBank accession numbers of the analyzed sequences. The four newly obtained sequences are of the '*Sphaeroplea*' clade.

### Alignment and phylogenetic analyses

Using 4SALE [40,41] with its ITS2 specific scoring matrix, we automatically aligned sequences and structures simultaneously. Sequence-structure alignment is available at the ITS2 database supplements page. For the complete alignment we tested for appropriate models of nucleotide substitution using the Akaike Information Criterion (AIC) as implemented in Modeltest [42]. The following PAUPblock was used for all maximum likelihood based phylogenetic analyses with PAUP* [43]: Lset Base = (0.2299 0.2415 0.2152) Nst = 6 Rmat = (1.4547 3.9906 2.0143 0.1995 3.9906) Rates = gamma Shape = 1.1102 Pinvar = 0.0931;. A maximum likelihood (ML) analysis was performed with a heuristic search (ten random taxon addition replicates) and nearest neighbour interchange (NNI) [44].

Maximum parsimony (MP) [45] was accomplished with gaps treated as missing data and all characters coded as "unordered" and equally weighted. Additionally, we clustered taxonomic units with neighbour-joining (NJ) [46] using maximum likelihood distances. Furthermore, with MrBayes [47] a Bayesian analysis (B) was carried out for tree reconstruction using a general time reversible substitution model (GTR) [48-50] with substitution rates estimated by MrBayes (nst = 6). Moreover, using ProfDist, a profile neighbour-joining (PNJ) tree [51,25] was calculated using the ITS2 specific substitution model available from the ITS2 Database. PNJ was also performed with predefined profiles (prePNJ) of all the clades given in Table 1.

**Figure 1:**
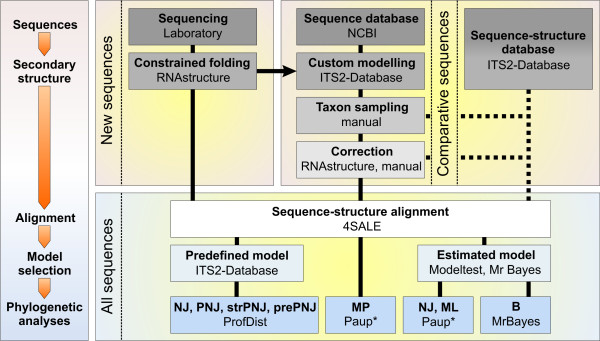
**Flowchart of the methods applied in this study**. Sequences were obtained from the laboratory and from NCBI and afterwards folded with RNAstructure [38] or custom modelling of the ITS2 Database [30-33]. An alternative way may pose to directly access sequences and structures deposed at the ITS2 Database. The sequence-structure alignment was derived by 4SALE [40]. Afterwards several phylogenetic approaches were used to calculate trees: NJ = neighbour-joining, PNJ = profile neighbour-joining, strPNJ = sequence-structure neighbour-joining, prePNJ = predefined profiles profile neighbour-joining, MP = maximum Parsimony, ML = maximum likelihood and B = Bayesian analysis.

For clade '*Scenedesmus*' two profiles were used for groups 'true *Scenedesmus*' (*Scenedesmus *except *S. longus*) and '*Desmodesmus*' (*Desmodesmus *and *S. longus*). We performed a sequence-structure profile neighbour-joining (strPNJ) analysis with a developmental beta version of ProfDist (available upon request). The tree reconstructing algorithm works on a 12 letter alphabet comprised of the 4 nucleotides in three structural states (unpaired, paired left, paired right). Based on a suitable substitution model [40], evolutionary distances between sequence structure pairs have been estimated by maximum likelihood. All other applied analyses were computed only on the sequence part of the sequence-structure alignment. For MP, NJ, PNJ, prePNJ and strPNJ analyses 1.000 bootstrap pseudoreplicates [52] were generated. One hundred bootstrap replicates were generated for the ML analysis. Additionally we used RAxML at the CIPRES portal to achieve 1.000 bootstraps with a substitution model estimated by RAxML [53]. All methods were additionally applied to a 50% structural consensus alignment cropped with 4SALE (data not shown). The individual steps of the analysis are displayed in a flow chart (Fig. 1).

## Results

### New ITS2 sequences

**Figure 2:**
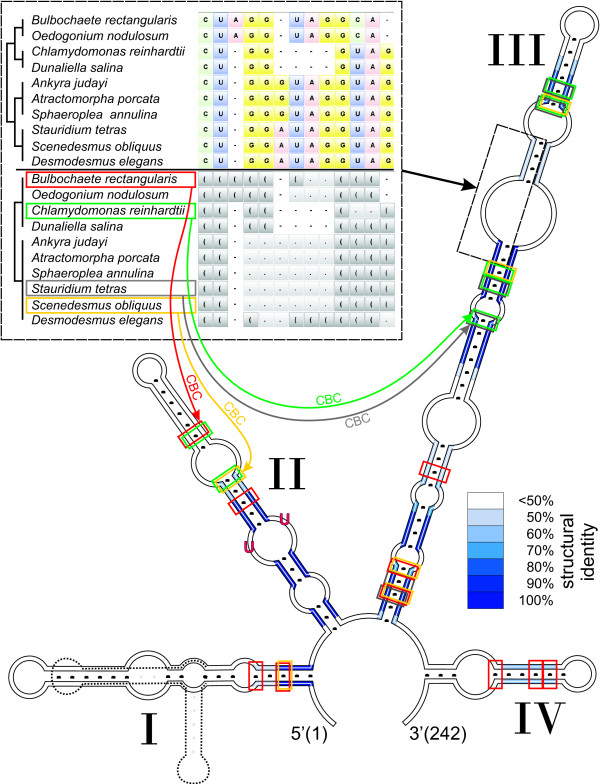
**ITS2 structure of *Sphaeroplea annulina*, degrees of conservation and structure alignment**. The structure of the internal transcribed spacer 2 of *Sphaeroplea annulina *shows the common four helices. Helix I is unbranched. Helix I of *Scenedesmus obliquus *with its branch is underlain in grey. The degree of conservation over the whole alignment is indicated in blue with different degrees of colour saturation. The structural consensus function of 4SALE [40] returns nucleotides on given percentages. In the upper left corner is the sequence-structure alignment of the conserved distal part of helix III showing a differentiation of the major clades with sequence and/or structure.

GenBank accession numbers for newly obtained nucleotide sequences are given in Table 1 (entries 1–4). The two ITS2 sequences of *Sphaeroplea annulina *(Roth, Agardh) strain SAG 377-1a and strain SAG 377-1e were identical and thus only the first one was used for further analysis. According to folding with RNAstructure, ITS2 secondary structures of the three newly obtained sequences did not exhibit any branching in their helix I (Fig. 2) as it is described for the 'core Sphaeropleales', i.e. helix I was more similar to those of the CW-group and the '*Oedogonium*' clade. Helix I of *Sphaeroplea *annulina was explicitly longer (9 nucleotides) than those of the other newly obtained algae. Due to this insertion, for *Sphaeroplea*, a branching pattern was enforceable, but would have lower energy efficiency. However, the additional nucleotides are not homologous to the insertion capable of making an additional stem (Y-structure) found in the '*Scenedesmus*' and the '*Hydrodictyon*' clade (approximately 25 bases).

### ITS2 sequence and secondary structure information

ITS2 sequence lengths of all studied species ran from 202 to 262 nucleotides (nt), 235 nt on average. The GC contents of ITS2 sequences ranged from 36.84% to 59.92%, with a mean value of 52.42%. The number of base pairs (bp) varied between 64 and 89 bp and averaged 77 bp. The cropped alignment (50% structural consensus) showed that 23% of the nucleotides had at least a 50% consistency in their pairings. Compensatory base changes (CBCs) as well as hemi-CBCs (all against all) range from 0 to 16 with a mean of 6.6 CBCs (Fig. 2). Sequence pairs lacking CBCs were exclusively found within the same major clade.

### Characteristics in a conserved part of alignment

In agreement with Coleman [28], the 5' side part near the tip of helix III was highly conserved including the UGGU motif [54,55,30], likewise the UGGGU motif in case of Chlorophyceae. We selected a part of the alignment at this position with adjacent columns (Fig. 2) to verify the suggested conservation. Having a closer look at this part of helix III, in our case, it showed typical sequence and structural characteristics for distinct groups. Studied species of the '*Oedogonium*' clade possess at position 3 in the selected part of the alignment an adenine and in addition at positions 3–5 paired bases. In contrast, the CW-group solely possessed three consecutively paired bases in this block, but not the adenine. A typical pattern for clades of the DO-group was a twofold motif of 3 bases: uracile, adenine and guanine at positions 7–9, which is repeated at positions 11–13. This could be a duplication, which results in a modified secondary structure. In addition, the '*core Sphaeropleales*' ('*Hydrodictyon*' clade and '*Scenedesmus*' clade) showed an adenine base change at position 6, compared to all other clades.

### Phylogenetic tree information

The PAUP* calculation applying maximum Parsimony included a total of 479 characters, whereas 181 characters were constant, 214 variable characters were parsimony-informative compared to 84 parsimony-uninformative ones.

**Figure 3:**
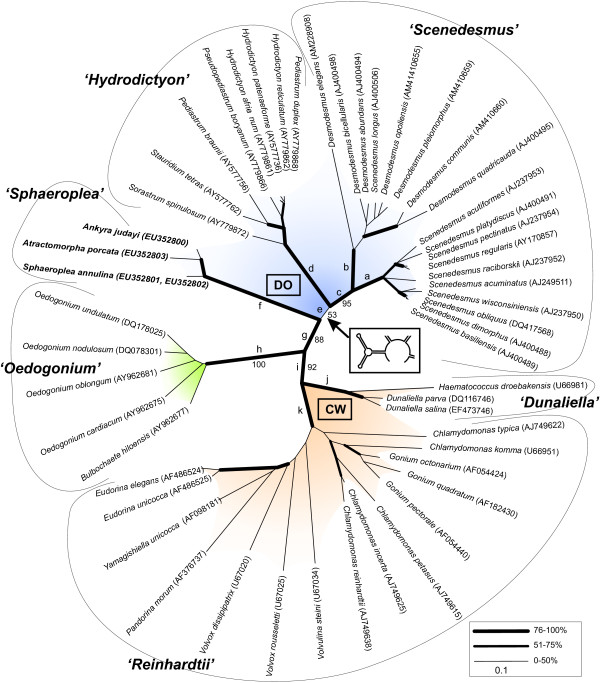
**Neighbour-joining phylogeny of the Chlorophyceae based on comparison of ITS2 rRNA sequences and structures**. The tree is unrooted, but the '*Oedogonium*' clade is most likely appropriate as outgroup [56]. Sequences of the '*Sphaeroplea*' clade were sequenced for this study and shown in bold letters. The phylogenetic tree is calculated by neighbour-joining with PAUP* [46,43] for an alignment with 52 taxa and 479 characters. The substitution model was set to TVM+I+G with parameters estimated by Modeltest [42]. Bootstrap values of basal branches are given for profile neighbour-joining with predefined profiles (ProfDist with ITS2 substitution model) [51,31]. Branch thickness is dependant of Bootstrap values calculated with four distance methods: neighbour-joining (PAUP*), neighbour-joining, complete profile neighbour-joining and sequence-structure profile neighbour-joining (all three ProfDist with ITS2 substitution model).

**Figure 4:**
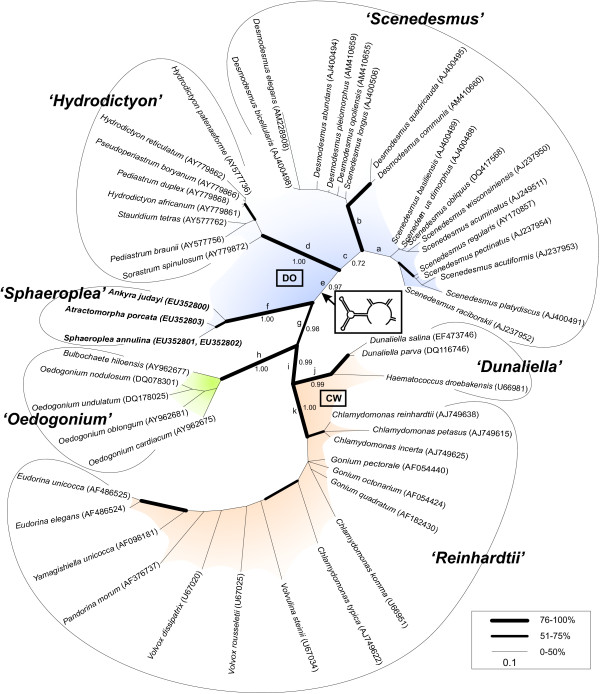
**Phylogeny of chlorophyte ITS2 sequences and structures based on distances of a Bayesian analysis**. The alignment contained 52 taxa and 479 characters. The suggested outgroup is the '*Oedogonium*' clade [56]. Sequenced species are shown in bold ('*Sphaeroplea*' clade). Substitution models and tree distances were calculated with MrBayes [47]. Posterior probabilities are shown for basal branches. Branch thickness is dependant of Bootstrap values calculated with maximum likelihood (PAUP* with TVM+I+G, RAxML) [42,53,43] and maximum Parsimony (PAUP*) (see legend). Resulting parameter of performing MP are L = 1231, CI = 0.4427, HI = 0.5573, RI = 0.7264, RC = 0.3216.

The resulting trees (Fig. 3 and 4, Table 2) of all performed analyses (NJ [PAUP* and ProfDist], PNJ, prePNJ, strPNJ, ML [PAUP* and RAxML], MP, B) yielded six major clades: the '*Dunaliella*', the '*Hydrodictyon*', the '*Oedogonium*', the '*Reinhardtii*', the '*Scenedesmus*', and the '*Sphaeroplea*' clade. All of them were separated and – except for the '*Scenedesmus*' clade – highly supported by bootstrap values of 83–100%, respectively by Bayesian posterior probabilities of 0.86–1.0.

**Table 2:** Bootstrap support values for basal branches of all methods applied.

Software		**ProfDist**				PAUP*			**MrBayes**	**RAxML**
				
Model		ITS2				Modeltest		-	Estimated	
				
Analysis		NJ	PNJ	prePNJ	strPNJ	NJ	ML	MP	B	ML
Nodes	a	99	95	100^1^	100	91	-	82	*0.86*	-
	b	96	96	100^1^	96	99	93	86	*1.00*	98
	c	88	88	95	88	90	-	63	*0.72*	-
	d	100	99	100^1^	100	100	92	100	*1.00*	96
	e	62	55	53	60	-	-	62	*0.97*	64
	f	100	100	100^1^	100	100	99	100	*1.00*	100
	**g**	**87**	**91**	**88**	**96**	**86**	**67**	**80**	***0.98***	**93**
	h	99	99	100^1^	99	100	100	100	*1.00*	100
	i	90	90	92	84	93	88	85	*0.99*	89
	j	97	98	100^1^	98	93	91	91	*0.99*	98
	k	97	96	100^1^	95	96	88	83	*1.00*	99
		
Figure		3					4			

The table supplements Fig. 3 and Fig. 4. Node "g" supports a monophyletic DO group and is printed in bold letters. Software used: ProfDist and PAUP*. Models of substitution: ITS2 = GTR with ITS2 substitution matrix, Modeltest: TVM+I+G with estimated parameters. Phylogenetic analysis: NJ = neighbour-joining, PNJ = profile neighbour-joining, prePNJ = profile neighbour-joining with predefined profiles, strPNJ = sequence-structure profile neighbour-joining, ML = maximum likelihood, B = Bayesian analysis (posterior probabilities), MP = maximum Parsimony. ^1^Predefined profiles for profile neighbour-joining.

The '*Hydrodictyon*' clade, the '*Scenedesmus*' clade and the '*Sphaeroplea*' clade form one cluster that was strongly supported by high bootstrap values of 67–96% (node "g"). The three clades composed the DO-group. The opposite cluster included the '*Dunaliella*' and the '*Reinhardtii*' clade, forming the CW-group. The '*Oedogonium*' clade was chosen as the outgroup [56]. Both clusters (CW-group and '*Oedogonium*' clade) were strongly supported by bootstrap values of 84–100% (nodes "i" and "h").

Except for the Bayesian analysis (least support for node "c"), all applied methods yielded node "e" as the weakest point within the basal (labelled) branches (Table 2), which presents the relationship between the '*Hydrodictyon*' and the '*Scenedesmus*' clade on the one hand and the '*Dunaliella*', the '*Oedogonium*', the '*Reinhardtii*' and the '*Sphaeroplea*' clade on the other hand. The phylogenetic tree resulting from neighbour-joining analysis by PAUP* (Fig. 3) did not support node "e" at all, but strongly supported the remaining labelled branches. The maximum likelihood analysis by PAUP* (Fig. 4) did not encourage node "e" either. Both maximum likelihood methods did not even support nodes "a" ('true *Scenedesmus*' compared to remaining clades) and "c" ('*Scenedesmus*' opposite to remaining clades). All other basal branches were supported by this method.

Varying neighbour-joining analyses by ProfDist (NJ, PNJ, prePNJ, strPNJ) supported all basal branches – except for the weakest node "e" (average support) – with very high bootstrap support values of 84–100%. The maximum Parsimony method gave average support (63 and 62%) for node "c" and "e" and high bootstrap values (80–100%) for the remaining basal clades. The Bayesian analysis offered posterior probabilities of 0.72 for node "c" and 0.86–1.0 for the remaining basal nodes. For further sister group relations see Fig. 3 and 4.

In comparison, the topology of the phylogenetic tree based on the 50% cropped alignment did not change, but the bootstrap support values were lower in all cases (data not shown).

## Discussion

The internal transcribed spacer 2 (ITS2) is required in ribosome biogenesis [57-59] and its gradual removal from mature rRNA is driven by its specific secondary structure [60,59].

Using three newly obtained ITS2 sequences from *Ankyra judayi*, *Atractomorpha porcata *and *Sphaeroplea annulina *(Sphaeropleaceae) in this study we aimed to pursue two consecutive questions concerning the phylogenetic relationships within Chlorophyceae. (1) What is the phylogenetic position of the newly sequenced algae relative to the 'core Sphaeropleales' and could the biflagellate DO-group be regarded as monophyletic? (2) How does the secondary structure of the new ITS2 sequences look like and is an autapomorphic feature of the secondary structure associated with the monophyletic DO-group?

Considering the question (1) Buchheim et al. [6] and Wolf et al. [23] approached the problem with 18S + 26S rDNA and 18S rDNA data, but the relationship between the 'core Sphaeropleales' and the Sphaeropleaceae remained unclear. However, in their studies, *Ankyra*, *Atractomorpha *and *Sphaeroplea *clustered in a monophyletic clade named Sphaeropleaceae. We confirm this '*Sphaeroplea*' clade with all three genera being strongly separated from other clades. As a result of a Bayesian analysis on a combined 18S and 26S rDNA dataset Shoup and Lewis [61] also found the Sphaeropleaceae as the most basal clade within the Sphaeropleales, but again the analysis lacked a strong backing. Beside these difficulties the 'core Sphaeropleales' were already shown to be monophyletic with high certainty [6,25,62,61,23].

The DO-group (Sphaeropleales including the '*Sphaeroplea*' clade) as emended by Deason et al. [10], for which the directly opposed basal body orientation and basal body connection features are verified [63-65], is now strongly supported by molecular phylogenetic analyses. There was already evidence of an extended DO-group [6,66,67], however, for some groups ultrastructural results are still lacking, and even though the collective basal body orientation and connection imply a monophyletic DO-group, until now no molecular phylogenetic analysis could show this with solid support [6,62,24,23]. We demonstrate for the first time with robust support values for the equivocal nodes that the 'core Sphaeropleales', the '*Sphaeroplea*' clade, and the Sphaeropleales are monophyletic.

Regarding question (2), for all structures of the '*Hydrodictyon*' and the '*Scenedesmus*' clade, helix I shows the typical branching (Y-structure). Initially, An et al. [68] proposed a secondary structure model with an unbranched helix I for ITS2 sequences of '*Scenedesmus*' clade members. Thereafter, van Hannen et al. [34] updated the model by folding the nucleotide sequences based upon minimum free energy and found a branched helix I as the most energetically stable option. The branching is result of an insertion of approximately 25 nucleotides capable of folding as an individual stem within the 5' end of the first helix. However, ITS2 sequence and secondary structure information of further '*core Sphaeropleales*' members, e.g. the '*Ankistrodesmus*' clade and the '*Bracteacoccus*' clade, lacks hitherto. In contrast, the Y-structure is absent within the '*Sphaeroplea*' clade and any other investigated group so far. Thus this feature is – contrary to our expectation – not an autapomorphic character for the biflagellate DO-group as a whole but for the 'core Sphaeropleales'.

Regarding future work, the resolution among the main clades of Chlorophyceae was statistically poorly supported in previous studies [68,15,6,23]. Pröschold and Leliaert [24] reviewed the systematics of green algae by applying a polyphasic approach, but did not yield a clear resolution regarding a sister taxon to the Sphaeropleales. Since they are not yet available, ITS2 sequences of chaetopeltidalean and chaetophoralean taxa could not be included in the present study and therefore the phylogenetic relationships between the main Chlorophyceae clades remain open. We recommend involving sequence and secondary structure information of chaetopeltidalean and chaetophoralean ITS2 sequences in future studies to find out if the monophyletic biflagellate DO-group could be further extended to a general monophyletic DO-group containing quadri- and biflagellate taxa. A genome-wide approach indicates that Sphaeropleales and Chlamydomonadales are sister taxa, however only a few organisms are included in this study [56]. An additional uprising question is when the Y has evolved within the 'core Sphaeropleales'. This could be resolved by inclusion of other members (e.g. *Bracteacoccus*) in further studies.

The two major reasons contributing to the robust results presented here are the change of the phylogenetic marker and the inclusion of secondary structure information. In contrast to previous phylogenetic work concerning Chlorophyceae, this study is based on the ITS2, which offers a resolution power for relationships from the level of subspecies up to the order level, because of their variable sequence but conserved secondary structure [26,30-33]. Hitherto commonly used markers in contrast are a lot more restricted. Using 4SALE [40] with implemented structure consideration, we could achieve for the first time a global simultaneously generated sequence-structure alignment (c.f. Fig. 1) yielding specific sequence and structural features distinguishing different algae lineages (c.f. Fig. 2).

## Conclusion

In summary, the powerful combination of the ITS2 rRNA gene marker plus a multiple global alignment based synchronously on sequence and secondary structure yielded high bootstrap support values for almost all nodes of the computed phylogenetic trees. Thus, the relationship of Sphaeropleaceae is here resolved, being a part of the Sphaeropleales representing the monophyletic biflagellate DO-group. Furthermore, we could elucidate a branched helix I of ITS2 as an autapomorphic feature within the DO-group. This feature could be found only in the '*Hydrodictyon*' and the '*Scenedesmus*' clade. Our results corroborate the presented methodological pipeline, the fundamental relevance of secondary structure consideration, as well as the elevated power and suitability of ITS2 in phylogenetics. For a methodological improvement it is suitable to ameliorate the alignment algorithm in further considering horizontal dependencies of paired nucleotides, and moreover in future ITS2 studies it is suggested to include sequence and secondary structure information of hitherto not regarded taxa to resolve the chlorophycean phylogeny.

## Authors' contributions

MW designed the study. FF determined the new sequences in the laboratory. BR implemented the strPNJ within ProfDist. TS and AK performed sequence analyses, structure prediction and phylogenetic analyses. TM developed the ITS2 sequence-structure substitution model and the ITS2 sequence-structure scoring matrix. TS, AK and MW drafted the manuscript. All authors contributed to writing the paper, read the final manuscript and approved it.
